# Engineering the Human Endometrial–Embryo Interface: Breakthroughs in 3D Uterine Models

**DOI:** 10.3390/biom16030383

**Published:** 2026-03-03

**Authors:** Jenna A. Douglas, Jordan Higgins, Dinasha H. Wimalasiri, Amy L. Winship, Harriet C. Fitzgerald

**Affiliations:** 1 Department of Obstetrics and Gynaecology, Monash University, Clayton, VIC 3168, Australia; jenna.douglas@monash.edu (J.A.D.); dinasha.wimalasiri1@monash.edu (D.H.W.); 2 The Ritchie Centre, Hudson Institute of Medical Research, Clayton, VIC 3168, Australia; 3 Monash Biomedicine Discovery Institute, Department of Anatomy and Developmental Biology, Development and Stem Cells Program, Monash University, Clayton, VIC 3800, Australia; jordan.higgins@monash.edu (J.H.); amy.winship@monash.edu (A.L.W.)

**Keywords:** endometrium, endometrial epithelial organoids, uterus, embryo implantation, placenta, trophoblast organoids

## Abstract

Three-dimensional (3D) organoid and co-culture models have emerged as transformative tools for studying human endometrial function, implantation, and placental development, overcoming key limitations of animal and two-dimensional in vitro systems. This review synthesises available information of recent advances in endometrial epithelial organoids (EEOs), trophoblast organoids (TBOs), and increasingly complex co-culture platforms incorporating stromal, vascular, and trophoblast compartments to model epithelial–stromal crosstalk, decidualisation, angiogenesis, and embryo implantation. Emerging developments include assembloid systems, synthetic and semi-synthetic extracellular matrices, and microfluidic organ-on-a-chip technologies that enable long-term culture, hormonal responsiveness, and patient-specific modelling. These approaches have recapitulated key features of the mid-secretory endometrium, placental villous architecture, trophoblast differentiation, and early implantation events while revealing disease-associated dysfunctions in conditions such as endometriosis, adenomyosis, polycystic ovarian syndrome, and endometrial cancer. Despite significant progress, current models remain limited by incomplete cellular diversity, polarity constraints, and challenges in fully modelling immune and vascular interactions. Collectively, emerging 3D organoid and co-culture systems provide physiologically relevant platforms to interrogate human reproductive biology, elucidate mechanisms underlying implantation failure and placental disease, and support the development of personalised therapeutic strategies to improve reproductive outcomes.

## 1. Introduction

Embryo implantation and pregnancy in humans will only occur when there is synchronicity in the development of the embryo and the endometrium to ensure a receptive state. The endometrium is the inner lining of the uterus and is made up of multiple cell types, including the luminal epithelium (LE), which is the initial site of embryo attachment and implantation, the glandular epithelium (GE), forming the endometrial glands and which is continuous with the LE, the stroma, which surround the glands, and the endothelial and immune cells. The human endometrium has two layers, an upper functionalis layer, which is shed during menstruation, and a lower basalis layer, which is adjacent to the myometrium, is retained during menstruation and is from which the functionalis reforms following menstruation. The endometrium must completely remodel to become receptive to embryo implantation, and, in particular, the endometrial epithelium produces factors that facilitate differentiation of the endometrial stroma into decidual cells and survival of the embryo [1,2]. Impaired endometrial function is associated with recurrent pregnancy loss, infertility and placental dysfunction. It is difficult to impossible to study the mechanisms underlying endometrial function and its role in infertility, embryo implantation and placental development in humans in vivo due to logistical and ethical constraints. The establishment of 3D models of the endometrium and early placenta now provide promising and exciting avenues for the greater understanding of endometrial biology and downstream development of potential treatments targeting endometrial infertility, associated diseases and placental dysfunction.

**Figure 1:**
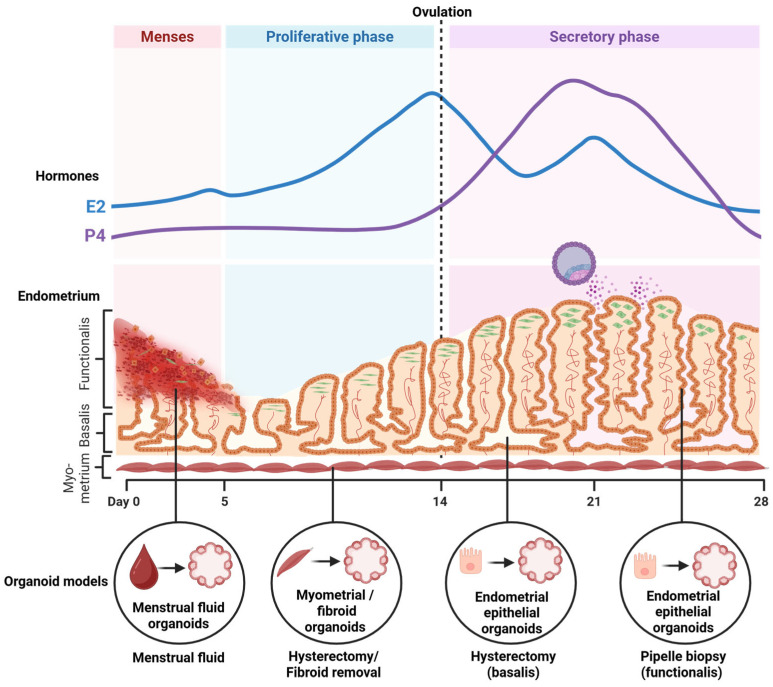
Cyclic regulation of the human endometrium across the menstrual cycle and corresponding organoid models. Schematic depiction of hormonal dynamics, endometrial architecture, and key cellular processes throughout the menstrual cycle. Top: circulating estradiol (E2, blue) and progesterone (P4, purple) levels across the menstrual, proliferative, and secretory phases. Middle: representative cross-sections of the endometrium illustrating breakdown and shedding during menses (day 0), regeneration and epithelial proliferation in the proliferative phase (days 5–14), and differentiation and glandular secretion in the progesterone-dominated secretory phase (days 14–28). Basalis and functionalis layers, stromal compartments, glands, and the myometrium are indicated. Ovulation occurs at the transition to the secretory phase, followed by stromal decidualisation and enhanced glandular secretory activity. Bottom: icons summarise phase- and tissue-specific sources of material to derive human organoids.

Endometrial remodelling is largely governed by the ovarian steroids estrogen and progesterone (Figure 1), whereby following withdrawal of these hormones, the functional layer of the endometrium is shed [1]. Endometrial regeneration is facilitated by endometrial stem/progenitor cells found in the basalis layer of the endometrium, and with rising estrogen, the endometrium proliferates during the proliferative phase [3,4,5]. Following ovulation, progesterone levels rise, and the endometrium differentiates during the secretory phase. During this phase, the endometrial glands become highly tortuous and secretory, secreting factors essential for embryo implantation [6]. Unique to mostly humans, the stromal cells undergo progesterone-induced spontaneous decidualisation, which is important for placental development and regulation of the maternal immune environment [7,8,9]. Animal models lacking endometrial glands are infertile and have impaired stromal cell decidualisation, highlighting the importance of endometrial glands for normal endometrial function, fertility and pregnancy establishment [10,11,12,13,14]. Endometrial gland secretions are disrupted in women with infertility, and dysfunction in stromal cell decidualisation is associated with endometriosis, recurrent pregnancy loss, implantation failure and preeclampsia [15,16,17,18,19,20,21,22,23,24].

Most animal models and 2D cell culture models of the endometrium do not recapitulate many aspects of endometrial hormone responsiveness, remodelling, embryo implantation and the crosstalk that occurs between the endometrium and embryo and the epithelium and stroma in humans in vivo. Conventional 2D cultures rely on studying the interaction of a trophoblast analogue and an endometrial analogue but fail to replicate the 3D architecture and cellular communication of implantation. Additionally, as placentation is an invasive process, effective models should be able to facilitate and support this. These limitations highlight the need for experimental models that better recapitulate what occurs in vivo. The sophisticated development of human endometrial epithelial organoids (EEOs) and trophoblast organoids as well as co-culture models of the different cell types (Figure 1) involved in implantation has provided essential platforms for the study of the fundamental reproduction processes as well as their potential use in treating infertility and reproductive pathologies. This review synthesises basic endometrial and placental biology with previously defined 3D cell culture models to provide a broad overview of current models and highlights existing gaps in modelling the embryo–endometrial interface. While there are existing reviews on each of these subjects individually [25,26,27,28,29,30], a single comprehensive review is yet to exist. This review synthesises the previous literature and expands on pitfalls of these models. Combining these topics into one review will allow researchers to better formulate research methods, particularly for disease modelling and its translational applications. Here, we review these 3D cell culture models and the most recent breakthroughs in modelling the human endometrial–embryo interface in vitro.

## 2. Methodology

This narrative review integrates the existing literature on the advances in three-dimensional cell culture models of the human endometrium and the early-stage embryo to highlight the potential in applying and/or combining many of these approaches to engineer the human endometrial–embryo interface in vitro in the future.

The PubMed and Google Scholar databases were searched from inception to January 2026. Papers reviewed include primary research, narrative reviews, and systematic reviews, with additional articles obtained from cited references of retrieved publications. A qualitative assessment of the literature was conducted, and no attempt to perform a systematic review was made to cover the broad interdisciplinary scope of the literature relevant to the sections discussed. This review covers a heterogeneous evidence base that compares key findings and methods from studies of three-dimensional culture models relevant to the study of human endometrial receptivity, embryo implantation and placentation, the clinical translatability of models pertaining to endometrial disease, and the future potential to build on recent advances.

## 3. Endometrial Epithelial Organoids

Human EEOs were established for the first time by two groups in 2017 who utilised knowledge gained from the intestinal stem cell organoid field, with modifications, to recapitulate endometrial epithelia in 3D (Figure 1) [31,32]. These EEOs were established from endometrial gland and epithelial fragments obtained by dissociating endometrial biopsies and decidua and were seeded within a mouse-derived basement membrane extract, Matrigel. The organoids were cultured in a defined serum-free medium (Table 1), limiting the constraints of batch variation in serums and the lack of hormone-responsiveness seen in traditional primary endometrial epithelial cultures [33]. These initial EEO studies highlighted the capacity for the organoids to be expanded, grown long-term and cryopreserved.

Importantly, EEOs mimic the epithelial cell types and hormone responsiveness of the human endometrium in vivo, which is essential to studying the complex remodelling and ability of the endometrium to become receptive to embryo implantation. EEOs are made up of both forkhead box A2 (FOXA2) (GE marker)-positive and -negative cells, suggesting that the EEOs contain a mixture of LE and GE cells [36]. Single-cell RNA sequencing and immunofluorescence of specific cell type markers has shown that EEOs also comprise ciliated and non-ciliated cells, secretory epithelium, stem-/progenitor-like cells, and proliferating epithelium [31,35,36]. The changes in the cell identities, gene and protein expression profiles of EEOs with treatment using menstrual cycle hormones, estrogen and progesterone indicates their similarity to those same changes seen within the endometrium in vivo during the proliferative and secretory phases of the menstrual cycle [31,32,35,36,40,41].

Endometrial glands and their secretions are essential for pregnancy establishment, providing nutrients to the developing fetus in the first trimester of pregnancy, and also impact stromal cell decidualisation [6,11,13]. Altered gland-derived products found in uterine fluid are associated with infertility in women [15,16,18,19]. The precise mechanisms governing endometrial secretions and their impact on embryo implantation and stromal cell decidualisation have been difficult to decipher given the lack of suitable models. The development of EEOs has now allowed for the comprehensive analysis of the epithelial secretome and comparisons between different patient cohorts [38,42,43]. Proteins secreted basolaterally from the endometrial epithelium likely interact in a paracrine manner with the surrounding stroma, and thus, investigating the conditioned media from EEOs containing the basolateral secretions has provided important insights into the regulation of stromal cell decidualisation [38]. Furthermore, EEO apical secretions are analogous with fluid found within the uterine cavity, which contains products impacting embryo implantation. Intra-organoid fluid containing apical epithelial secretions was collected from EEOs derived from patients with primary infertility and fertile women and was found to be altered between these cohorts [42]. Indeed, fertile EEO intra-organoid fluid improved the adhesion of human trophoblast progenitor cell spheroids to epithelial cells compared to fertile extra-organoid fluid (basal secretions) and infertile intra-organoid fluid [42]. These studies indicate that EEOs are essential for the study of patient-specific endometrial secretome, providing important information linking altered endometrial epithelial function and infertility in women.

**Figure 2:**
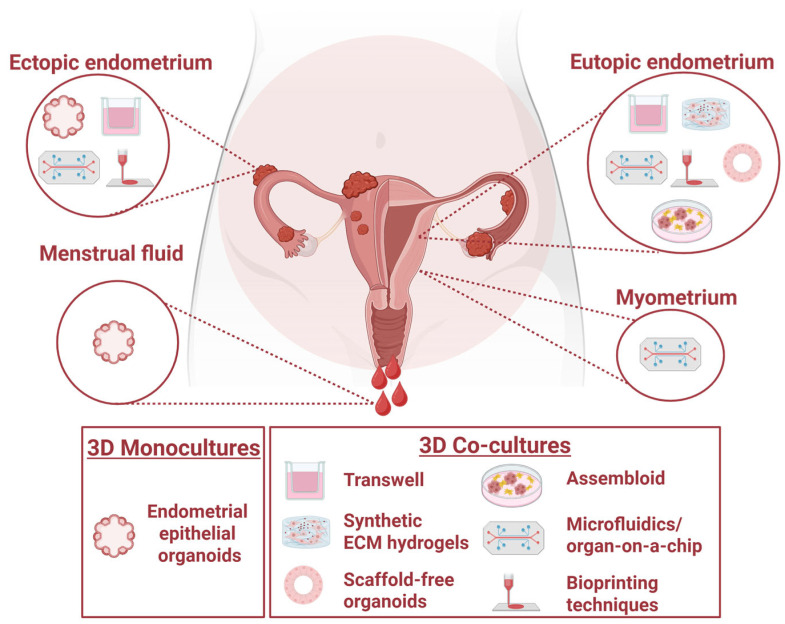
Sources of human endometrial tissues and contemporary 3D in vitro model systems used to study endometrial biology and disease. Schematic representation of key tissue sources—including ectopic endometrium, eutopic endometrium, menstrual fluid, and myometrium—used for generating advanced in vitro models. Expanded panels depict example 3D culture approaches derived from these tissues. 3D monoculture systems include endometrial epithelial organoids. 3D co-culture systems incorporate multiple cell types and microenvironments using platforms such as Transwell inserts, synthetic extracellular matrix hydrogels, scaffold-free organoids, assembloids, microfluidic/organ-on-a-chip devices, and bioprinting techniques. Together, these approaches recapitulate structural and functional features of human endometrium and enable mechanistic investigation of healthy and diseased states, including endometriosis.

Advances in EEO culture and utilisation include reduced invasiveness of endometrial tissue collection and improvements in the extracellular matrix (ECM) of which EEOs are cultured and its recapitulation of the endometrial ECM environment (Table 2). Menstrual fluid contains menstrual tissue, which can be collected non-invasively via a menstrual cup. Epithelial fragments have been isolated from menstrual fluid tissue and cultured as menstrual fluid organoids (MFOs), showing similar growth rates and hormone responsiveness to that of EEOs (Figure 2) [44,45]. Importantly, menstrual fluid content appears similar between cycles of the same patient, demonstrating its reliability in being a robust indicator of the endometrial environment, and it is a non-invasive method to assess the endometrial environment [44,45]. MFOs present a promising avenue for a targeted, personalised medicine approach to identifying suitable treatments for endometrial-related diseases and pathologies. Most EEO models utilised Matrigel, which is not reflective of the human endometrial ECM, exhibits batch-to-batch variation and does not support stromal cell growth, which is necessary for endometrial–stromal co-cultures (Table 2) [26,27]. Given that current standard matrices are prone to degradation, a spinning bioreactor was recently shown to support long-term 3D culture of EEOs by providing sustained suspension, extending the culture period beyond 28 days to mimic a 28-day menstrual cycle when treated with steroid hormones [39]. However, this is yet to be achieved in co-culture with other cellular compartments. Recent studies have developed and utilised different matrices, including decellularised endometrium made into a hydrogel, collagen matrices and fully synthetic polyethylene glycol (PEG) hydrogels mimicking the endometrial ECM [46,47,48,49,50]. Advancements in development of a representative endometrial ECM is essential for studying endometrial tissue remodelling and the direct interactions between the epithelium and stroma and epithelium and implanting blastocyst.

## 4. Three-Dimensional Co-Culture Models of the Human Endometrium

### 4.1. Three-Dimensional Co-Culture Models of Epithelial-Stromal Interactions

Evidence from gland-knockout animals has shown that the absence of endometrial glands leads to impaired decidualisation and infertility [11,12,13]. Although in vivo models have established that glands are a requirement for successful stromal decidualisation, studying human endometrial gland–stromal interactions and maternofetal interactions in vivo is not feasible. To recapitulate the human endometrium accurately, in vitro co-culture systems that capture paracrine mechanisms between epithelial and stromal cells are required. Knowledge of epithelial–stromal crosstalk has previously relied on cell-type-specific receptor-knockout mouse models [55], reflecting limitations of earlier in vitro models.

An ‘assembloid’ co-culture model containing EEOs and stromal cells revealed close resemblance to the mid-secretory native endometrium and also demonstrated that co-culturing with blastocysts can recapitulate implantation failure pathologies by modulating decidual senescence (Figure 2) [47]. However, this model was limited by a relatively short-term culture due to the collagen enriched ECM scaffold used. As such, co-cultures combining endometrial epithelial and stromal cells in a Matrigel or collagen scaffold have been unsuitable, lacking expandability and biobanking ability [9,53,56]. Matrigel has a poorly defined chemical composition of ECM proteins and growth factors, with batch-to-batch variability. Successful establishment of a human endometrium-derived decellularised ECM hydrogel has shown to adequately support the growth of human organoid cultures, offering a potential alternative to Matrigel- and collagen-based matrices for improving the 3D organoid culture model (Figure 2) [49]. However, its applicability for co-culturing EEOs and stromal cells remains to be determined.

EEOs derived from estrogen receptor alpha (ESR1)-null mice spontaneously formed multilayered lobular structures in culture [52]. These organoids recapitulated key features of cervicovaginal epithelium and expressed basal cell markers, including Trp63, Krt5 and Krt14. To interrogate epithelial–stromal interactions, mouse EEOs were co-cultured with stromal fibroblasts in transwell systems using Cultrex as a three-dimensional extracellular matrix. This approach demonstrated a pivotal role for stromal cells in regulating lineage plasticity within the uterine epithelium [52]. Cultrex basement membrane extract (BME), composed primarily of laminin and collagen, is derived from murine tumours and is subject to batch-to-batch variability, although it exhibits biochemical and mechanical properties comparable to Matrigel (Table 2). To overcome limitations associated with matrix-derived scaffolds, Wiwatpanit et al. (2019) established a scaffold-free three-dimensional multicellular organoid co-culture model of human primary endometrial epithelial and stromal cells [57]. When maintained in micromolded agarose gels, these organoids reproduced hallmark morphological features of endometriotic lesions, characterised by centrally localised stromal cells surrounded by polarised epithelial cells lining the outer surface.

Other materials such as gelatin methacryloyl (GelMA) [50,58] and collagen [53] have been used as scaffolds for the bioengineering of endometrial tissue (Table 2). Using a collagen-based scaffold, Abbas et al. [53] established a co-culture model in which an EEO-derived epithelial monolayer was seeded with its apical surface exposed while stromal cells were embedded within the underlying matrix. The resulting luminal-like epithelial layer demonstrated functional hormone responsiveness. This co-culture system provides a platform for further incorporation of additional cell types, enabling more comprehensive recapitulation of native endometrial tissue architecture and function.

Replacement of naturally derived hydrogels with chemically engineered, modifiable synthetic or semi-synthetic hydrogels provides the hope of enhancing the physiological relevance of the organoid model by enabling epithelial–stromal crosstalk (Table 2) [46,50]. A recent co-culture model using a modified synthetic polyethylene glycol (PEG)-based ECM demonstrated the ability to culture both human EEOs and endometrial stromal cells long-term while sharing the same matrix, allowing the model to physiologically and morphologically mimic cycle phase changes in response to hormones as in vivo (Figure 2) [46]. The PEG-based co-culture model also revealed a dysregulation in key hormone-mediated functions in the disease context via epithelial–stromal interactions, such as stromal cell decidualisation. The diseased state was induced by IL-1β, a key modulator of physiological inflammation in the eutopic endometrium and a known upregulated marker in the pathophysiological environment of endometriosis lesions [46]. Salisbury et al. (2024) also demonstrated the ability to successfully co-culture primary human endometrial stromal cells and EEOs with hormonal responsiveness using a semi-synthetic photo-cross-linked GelMA hydrogel combined with bioactive properties of natural hydrogels [50]. With these matrices that more closely recapitulate the structure and function of the native endometrium than currently commercially available hydrogels, these studies represent critical steps in modelling direct endometrial epithelial–stromal crosstalk and investigating other cellular mechanisms within a 3D environment in the context of reproductive failure and associated gynaecological disorders.

### 4.2. Three-Dimensional Co-Culture Models with Maternal Vasculature

The successful establishment of pregnancy requires an orchestrated process that involves the adhesion of the embryo to the receptive endometrial epithelium followed by controlled trophoblast invasion into maternal tissue. Concurrent vascular remodelling and angiogenesis at the implantation site is essential for establishing an adequate maternal–fetal nutrient exchange. Disruptions to the process of angiogenesis during this critical time of trophoblast implantation can lead to critical pregnancy complications.

To remodel the dynamic environment of the endometrium, microfluidic devices have been used to create the ‘organ-on-a-chip’ model incorporating live cells cultured under fluid flow [54]. Gnecco et al. (2017) devised a dual-chamber microfluidic device which allowed for the long-term co-culture of primary vascular endothelial cells derived from human umbilical vein and endometrial stromal cells [59]. Park et al. (2022) engineered a human implantation-on-a-chip model using a triple-chamber system, which involved a fetal chamber comprising primary extravillous trophoblast (EVT) cells and a maternal chamber comprising stromal and endothelial cells, separated by a matrix-filled chamber, to investigate trophoblast invasion at the materno-fetal interface [60]. Unlike this model, Dai et al. (2025) included an epithelial component by combining endometrial epithelial organoids, stromal cells and endothelial cells within a Matrigel and fibrin-based hydrogel matrix to develop a vascularised triple-cellular endometrial complex in a microfluidic chip, called an endometrium-on-a-chip (eCHIP) [61]. The eCHIP was hormonally responsive and suggested that increasing levels of WNT7A and WNT5A are responsible for the stimulation of vascular neogenesis. With the addition of smooth muscle myocytes to an endometrial stromal and epithelial cell microfluidic co-culture, Busch et al. (2024) developed a patient-derived tri-culture spheroid model of the uterine wall [62]. This model, in its microfluidic environment, was established by sequential seeding of individual and combined cell fractions in Matrigel. The spheroid formed had varying morphology; however, epithelial cells were localised to the outer area of the spheroid [62]. Similarly, a patient-derived vascularised endometrium-on-a-chip (EoC) incorporating primary EEOs and endometrial stromal cells, with a vascular channel containing human umbilical vessel endothelial cells, was established recently and shown to successfully capture the microenvironment of the native endometrium [63]. Using this EoC model, Lee et al. (2025) developed a clinically relevant scoring system for endometrial receptivity, enabling a personalised analysis of endometrial health and implantation potential across a heterogeneous patient population [63]. The development of integrated in vitro models incorporating diverse endometrial cell fractions enables a more comprehensive reconstruction of the endometrial architecture and facilitates evaluation of the cellular crosstalk that occurs between its distinct layers and at the materno-fetal interface for the investigation of endometrial receptivity and implantation.

### 4.3. Modelling the Luminal Epithelia

During the mid-secretory phase of the endometrium, luminal epithelial (LE) cells undergo morphological and molecular changes to enhance receptivity for blastocyst attachment preceding implantation. Impediments to these changes may drastically impact receptivity and are deemed a key contributor to implantation failure.

Primary human endometrial epithelial cells (pHEECs) isolated from endometrial biopsies can be seeded onto a culture plate as a monolayer to mimic the LE and investigate adhesiveness to an implanting embryo. Evans et al. [64] produced an ‘embryo–endometrial’ adhesion model using 3D trophoblast spheroids and a pHEEC monolayer mimicking the LE to predict a ‘non-receptive’ endometrium [64]. This model revealed significantly reduced trophoblast spheroid adhesion to pHEECs from infertile patients compared to fertile patients. Using a similar adhesion model in humans and mice, Zhou et al. (2021) revealed that knockdown of Notch ligand Jagged 1 (JAG1), previously shown to be localised to the LE, in the pHEECs significantly reduced trophoblast spheroid attachment and expression of receptivity markers such as leukaemia inhibitory factor receptor (LIFR), secreted phosphoprotein 1 (SPP1) and insulin-like growth factor-binding protein 1 (IGFBP1), corroborating its applicability as a receptivity model [65]. Despite this, both studies are limited by the assessment of initial attachment and adhesion stages of implantation in the absence of in vivo-like epithelial apicobasal polarity and endometrial cell–cell interactions. This prompts the need for a 3D endometrial model incorporating EEOs with established apicobasal polarity, ideally involving other cellular compartments, to participate in such studies.

EEOs can be used to produce epithelial cell monolayers, expressing ciliated epithelial cell marker acetylated α-tubulin, to participate directly in the deposition of an implanting blastocyst-like model [66]. While EEOs have revolutionised 3D in vitro modelling of endometrial epithelia and have proven their ability to recapitulate features of uterine glands in vivo [31,34,36], they remain a mixed epithelial cell population consisting of both GE and LE cells [32,36], with a sizeable proportion of these cells expressing glandular epithelial marker FOXA2 [36,66]. Additionally, the apicobasal polarity of EEO-derived monolayers is yet to be characterised. Another approach has been proposed to stimulate the differentiation and formation of ciliated LE cells with secreted products from decidualised stromal cells, such as prolactin [31], since the stroma underlying the luminal surface interacts with the LE in vivo. Using a transwell insert, Tian et al. (2023) showed that they could generate ciliated cells using an air–liquid interface culture system for endometrial assembloids (ALI-EnAo) to form in vivo-like luminal epithelial structures [51].

Despite these approaches, no published studies to date have successfully isolated human LE from GE and defined their precise differential roles in receptivity and implantation in vitro. This is essential to uncover the underlying defective mechanisms that contribute to endometrial dysfunction, which in turn leads to implantation failure across various gynaecological pathologies. A deeper understanding of these mechanisms will enable the development of targeted therapeutic strategies to treat diseases associated with infertility and improve reproductive outcomes.

## 5. Modelling Endometrial Dysfunction in Gynaecological Disease

The importance of endometrial glands to achieve successful pregnancy is well established from in vivo mouse models. Mouse models lacking gland-derived factors forkhead box A2 (*Foxa2*), Leucine-rich repeat-containing G-protein coupled receptor 4 (*Lgr4*), wingless-type family member 4 (*Wnt4*) and leukaemia inhibitory factor (*Lif*) demonstrated either an absence or reduced presence of glands, as well as impaired decidualisation and implantation [67,68,69,70].

Despite this knowledge, most animal models lack suitability for the study of dysfunctional processes involved in endometrial receptivity and embryo implantation associated with gynaecological disease due to their lack of a menstrual cycle and spontaneous steroid hormone-driven stromal cell decidualisation that occurs within the human endometrium [71,72]. These limitations have forced major challenges in modelling human gynaecological and female reproductive disorders—particularly their impact on human endometrial function. Consequentially, there is a need to establish and refine a three-dimensional co-culture model of the endometrial–embryo environment to understand and define the impact of gynaecological disorders on endometrial function and blastocyst implantation at a cellular and molecular level.

Endometrial disorders represent a major gynaecological problem and a significant cause of infertility. EEOs have been utilised to develop 3D models of endometrial dysfunction across a range of gynaecological pathologies and malignancies associated with infertility and implantation failure such as endometriosis (ectopic and eutopic endometria) [34,73], adenomyosis [74,75,76,77], uterine fibroids [78,79] polycystic ovarian syndrome (PCOS) [80], endometrial cancer [34,81,82] and pre-cancerous endometrial hyperplasia [34].

### 5.1. Endometriosis

Endometriosis is a chronic, debilitating gynaecological disorder which affects ~10% of women [83] and is defined by the presence of endometrial-like tissue fragments outside of the uterus in the pelvic cavity, forming ectopic endometrial lesions [25]. These ectopic lesions remain hormonally responsive and undergo cyclical changes in response to fluctuations across the menstrual cycle, such as inflammation, bleeding and the formation of adhesions and scar tissue.

Infertility affects 30–50% of endometriosis sufferers [84], with no fully effective therapies currently available. While in vitro fertilisation (IVF) remains one of the most successful available forms of assisted reproductive technologies (ARTs), implantation and pregnancy rates are significantly lower in women with endometriosis compared to women with other causes of infertility undergoing IVF [85,86,87]. The secretory-phase eutopic endometrium from patients with moderate–severe endometriosis has an altered gene expression profile compared to patients without endometriosis [88], suggesting impaired hormone responsiveness within the eutopic endometrial epithelia. Despite this, the mechanisms of endometriosis-associated infertility remain largely unknown.

Given that endometriosis occurs spontaneously only in humans and some menstruating non-human primates, endometriosis can be induced by transplantation of endometrial tissue in the peritoneal cavity of rodents and non-human primate models, leading to ectopic lesions that mimic endometriosis [89,90]. However, endometriosis-like lesions within these models have been unable to form deep infiltrating endometriosis (DIE) lesions, and these models are typically short-term. Studying the impact of endometriosis on human endometrial function, endometrial receptivity and embryo implantation in these models introduces practical, ethical, and economical challenges. Despite the advantageous use of cell lines to overcome this, they fail to replicate the phenotypic heterogeneity that exists. Patient-derived human primary cells from eutopic endometrium and ectopic lesions provide better phenotypic and genotypic similarity to the native tissue of origin. With the limited ability of animal models to accurately recapitulate human disease pathophysiology, robust and physiologically relevant three-dimensional in vitro models of endometriosis, such as ectopic-lesion-derived organoids, have been established to capture patient heterogeneity, identify novel disease mechanisms and improve drug development [34,73,91,92].

Boretto et al. (2019) [34] were the first to successfully establish patient-derived EEOs from the ectopic lesions (ECT-O) as well as the eutopic endometrium (EUT-O) of endometriosis patients (Figure 2). ECT-O exhibited histological similarities to the primary in vivo lesion and revealed more variation in gene expression clustering likely due to the different lesion type and stage classification [93], reflecting the high level of heterogeneity between patients [34]. Transcriptomic analysis of ECT-O revealed endometriosis-stage-specific gene expression associated with altered signalling pathways, such as PI3K-AKT and WNT, when compared to organoids derived from healthy endometrium. Altered gene expression patterns of progesterone receptor (*PGR*) and reduced expression of progesterone-regulated gene *PAEP* [34] were also observed, consistent with previous findings indicative of a P4-resistant and E2-dominant phenotype in patients with endometriosis [73,88]. While there was greater emphasis on lesion-derived organoids in this study, comparing EUT-O with organoids derived from normal endometrium revealed a downregulation of BMP and activin membrane-bound inhibitor (*BAMBI*) and an upregulation of Growth Differentiation Factor 11 (*GDF11*) in EUT-O, suggesting an upregulated transforming growth factor (TGF)-β signalling pathway within the eutopic endometrium of patients with endometriosis [34], which is likely to hinder endometrial receptivity based on clinical findings [94]. *BAMBI* inhibits TGF-β/BMP signalling and facilitates WNT signalling, suggesting an indirect role in the receptive transformation and differentiation of the endometrium and embryo implantation [94,95,96]. Notably, TGF-β members have previously been associated with endometriosis pathogenesis and likely serve as biomarkers for disease severity [97]. Trophoblast differentiation and invasion, which is critical for establishing the maternal–fetal interface during embryo implantation, is also regulated by TGF-β signalling [98]. While Zhang et al. (2025) and Gunther et al. (2025) successfully produced ECT-O from endometrioma and ECT-O from all lesion subtypes, respectively, to validate their feasibility as a pre-clinical model [73,92], no comparisons were made between EUT-O and EEO from healthy patients in this study to make any inferences concerning receptivity- or implantation-related mechanisms as the larger focus of this review.

Luddi et al. (2020) demonstrated that eutopic EEOs from endometriosis patients (EndORG) had decreased Glycodelin-A (GdA) protein expression levels compared to EEOs from healthy patients [37], replicating existing data from endometrial tissue biopsies in the proliferative phase and implantation window [99], confirming that this model recapitulates the in vivo disease phenotype. Despite this, their feasibility for clinical use has remained uncertain.

EEOs derived from the eutopic endometrium and ectopic lesions of endometriosis patients have the potential of representing the high degree of variability and phenotypic heterogeneity between individuals. Gnecco et al. (2023), on the other hand, proposed a possible ‘lesion’ model by combining EEOs and stromal cells from the endometrium of healthy patients within a modified synthetic PEG hydrogel in the presence of inflammatory marker interleukin 6 (IL-6) [46]. While this model was not derived from the primary lesions themselves, this study was the first to successfully co-culture primary patient-matched EEOs and stromal cells using a modified synthetic matrix, and it emphasised the importance of using 3D co-cultures to assess epithelial–stromal interactions, which will enable us to uncover novel mechanisms underlying endometriosis lesion formation and endometrial dysfunction in endometriosis-related infertility. Enhancing the patient-derived EEO model to include important cell–cell interactions, such as those described earlier in this review, represents substantial potential in defining the mechanisms by which endometriosis impacts fertility.

Using EEOs derived from women with primary infertility, Zhou et al. showed that infertile EEOs had dysregulated apical secretions in the intra-organoid fluid (IOF) in response to steroid hormones, which altered trophoblast cell adhesion compared to women with normal fertility [42]. By treating a primary endometrial stromal cell culture with select proteins identified within the basolateral secreted proteome of EEOs treated with menstrual cycle hormones, Fitzgerald et al. (2023) were the first to demonstrate that the basolateral protein secretions alter stromal cell decidualisation, suggesting that these proteins may be coordinating decidualisation in normal physiological conditions [38]. These approaches are yet to be applied to patients with other gynaecological disorders such as endometriosis, a condition associated with disordered decidualisation [21]. Collectively, this suggests that EEOs provide a valuable tool to uncover mechanisms for aberrant endometrial cell–cell crosstalk hindering endometrial receptivity and implantation in patients with endometriosis or other gynaecological pathologies.

### 5.2. Adenomyosis

Adenomyosis is a gynaecological condition characterised by the infiltration of endometrial glands and stroma into the myometrium, resulting in symptoms such as abnormal uterine bleeding, which can lead to severe menstrual cramps and pelvic pain, and even anaemia, in many patients [100]. In some cases, the adenomyotic endometrial tissue continues responding to hormonal fluctuations as it thickens, breaks down, and bleeds during each menstrual cycle, enabling the uterus to become enlarged and tender [100].

While the exact cause of adenomyosis remains unclear, uterine trauma, elevated estrogen levels, and stem cells have been proposed as likely contributing factors [101,102]. Historically, evidence suggested that a perturbed boundary between the basal endometrial layer and myometrium due to pregnancy enables the endometrial cells to invade the uterine muscle in parous women [101]; however, this theory does not explain the disease aetiology in nulliparous women, particularly those reported to suffer from adenomyosis-related infertility [103,104].

While a human cell culture model to investigate the cause of adenomyosis was yet to be achieved until recently, EEOs derived from endometrial biopsies of adenomyosis patients have been established as a pre-clinical model to study impaired implantation and poor pregnancy outcomes associated with adenomyosis [74]. The EEOs were treated with hormones to induce either secretory-phase or gestational-phase differentiation. Gene expression of secretory-phase and endometrial receptivity markers *SPP1*, *PAEP*, *LIF* and *17βHSD2* were upregulated in both secretory and gestational-adenomyosis-derived EEOs compared to control EEOs, suggesting a possible mechanism for impaired implantation in these patients [74]. Following this study, Juárez-Barber et al. (2024) performed a transcriptome analysis of adenomyosis-derived EEOs [77]. Differentially expressed genes (DEGs) identified in adenomyotic mid-secretory-phase EEOs included upregulation of Olfactomedin 1 (*OLFM1*) [84], which was previously associated with a non-receptive endometrium [105,106,107]. Based on these findings, a pathway analysis of DEGs predicted a dysregulation of signalling pathways associated with poor reproductive outcomes in patients with adenomyosis. For example, upregulated high-mobility group box 1 (HMGB1) signalling was associated with reduced adhesion in epithelial cells of patients suffering from recurrent implantation failure (RIF) [108], and a downregulation of inhibitor of DNA binding 1 (ID1) signalling pathway was detected and associated with impaired decidualisation due to aberrant stromal cell differentiation [109]. Despite this, additional in vivo studies are required to validate these proposed mechanisms for endometrial dysfunction in patients suffering from adenomyosis-related infertility.

Epithelial–stromal assembloids from adenomyosis patients have recently been generated as a more complex model of impaired endometrial receptivity associated with adenomyosis [75], where adenomyosis-derived assembloids exhibited dulled hormone responsiveness and reduced leukaemia inhibitory factor (LIF) expression in the stromal compartment, which is associated with implantation failure in patients with unexplained infertility [110]. More recently, Xu et al. (2026) were the first to build this model further by establishing a 3D multicellular assembloid that combines EEOs with stromal cells and myometrial smooth muscle cells from both eutopic and ectopic endometria of adenomyosis patients [76], identifying aberrant WNT signalling as a possible disease-associated mechanism. The transcriptional profiles in this assembloid model replicated pathological features of adenomyosis when compared with in vivo data, suggesting that this model may recapitulate adenomyosis-related endometrial dysfunction and enable the identification of novel mechanisms as therapeutic targets. Ultimately, assembloid models of eutopic and ectopic endometria offer promising opportunities to develop targeted therapies that will restore endometrial function and/or reduce lesion development as well as to improve clinical management of this condition in the future.

### 5.3. Polycystic Ovarian Syndrome (PCOS)

Polycystic ovarian syndrome (PCOS) is a common complex endocrine disorder which affects ~11–13% of women worldwide and is characterised by metabolic and reproductive disruptions, including infertility associated with implantation failure due to endometrial abnormalities [111]. EEOs obtained from patients with PCOS have been recently established, comparing two weight phenotypes (overweight/obese and lean) with Body Mass Index (BMI)-matched controls [80]. While Luyckx et al. showed that control EEOs have the same physiological responses to sex hormones as reported previously [31,34], they showed that this EEO model was able to capture distinct endometrial epithelial abnormalities in both PCOS weight categories. For example, PCOS-EEOs from both weight categories had increased expression of inflammation-related genes, such as *Oncostatin M* Receptor (*OSMR*) and *Intercellular Adhesion Molecule 1* (*ICAM1*), compared to control EEOs. Only one transcriptomic study exists which reports on PCOS endometrial epithelia from BMI-matched controls, demonstrating that *ICAM1* is also dysregulated in the endometrial epithelia of patients with PCOS [112] and providing translational relevance of the PCOS-EEO model. Both weight categories of PCOS-EEOs showed reduced expression of receptivity-related genes *PAEP* and *LIF*, similarly to other endometrial disorders [34,113], which may suggest shared dysfunctional pathways across gynaecological conditions. A recent transcriptomic study of the endometrium also demonstrated aberrant *PAEP* expression in the luminal epithelia of patients with PCOS [114], further confirming that PCOS-EEOs recapitulate in vivo disease phenotype. Furthermore, the similar transcript findings between BMI cohorts of PCOS-EEOs also suggest that inflammation, and potentially other dysregulated processes, is a hallmark of PCOS status rather than BMI [80]. This study provides the first evidence for the potential use of PCOS-derived EEOs to define PCOS-related endometrial dysfunction that may be causing altered endometrial receptivity, implantation failure and subsequent pregnancy complications in women with PCOS.

### 5.4. Endometrial Cancer

Endometrial cancer (EC), particularly endometrioid adenocarcinoma, represents the most common malignancy of the uterus, accounting for approximately 80% of uterine cancer diagnoses. An established risk factor for the development of hormone-sensitive endometrial cancer is prolonged and unopposed exposure to estrogen, which disrupts endometrial homeostasis and promotes uncontrolled cellular proliferation [115]. Fertility-sparing approaches are required to enable a successful pregnancy and possible remission in pre-menopausal patients with endometrial carcinoma, either without or before the need for a complete hysterectomy.

The clinical relevance of cancer cell lines for chemotherapeutic screening has been a subject of debate previously [116], with drug responses of many cell lines failing to be recapitulated in clinical trials [117]. For the same reason, no reliable approaches have been developed to predict and identify subsets of patients who will effectively respond to endocrine-based treatments.

Patient-derived tumour organoids (PDOs), also referred to as PDTOs or EC-Os, from human endometrial carcinoma biopsies represent a rapidly growing area of endometrial cancer research as an enhanced pre-clinical model for gynaecological tumours that can be used for drug screening [31,34,81,82,118,119,120,121,122]. Notably, Turco et al., Girda et al. and Maru et al. first demonstrated that PDOs recapitulate the tumour of origin by retaining similar morphological and histological features of the original tumour, including dense structure and isolated clusters of cells with nuclear abnormality. PDOs also expressed EC-associated markers such as ER, PR, p53 and/or proliferative marker Ki67 to the original tumour [31,82,118]. Boretto et al. (2019) [34] comprehensively demonstrated genetic and mutational similarities between endometrial cancer-derived PDOs (EC-Os) and the tumour from which they were derived, identifying mutations in genes commonly seen in EC, including *PIK3CA*, *TP53*, *PTEN* and *ARID1A*, which corresponds to findings from subsequent studies [118,120,121,123]. Chromosome 17 gains and PI3K-AKT signalling pathway hyperactivation, which are commonly observed in EC [124], were also identified, corroborating their ability to model disease- and type-associated gene expression [34]. The feasibility of PDO cultures for the application of cancer drug sensitivity testing was first demonstrated by Girda et al. (2017), showing that growth of most drug-treated PDOs was inhibited [82]. Boretto et al. revealed that EC-Os exhibited patient-specific drug responses, providing further evidence for their amenability to personalised drug screening [34]. Using PDOs from four patients, Chen et al. validated drug candidate MI-136 as a potential inhibitor and identified the menin–HIF axis as a novel mechanism for endometrial cancer [119]. Most recently, Vaccarella et al. (2025) [120] compared drug responses of PDOs to the oncological outcomes of the patients from which they were derived. Of the five PDOs, 100% mirrored the patients’ responses to therapy. However, the potential value of the PDO model for clinical decision making requires clinical trial evaluation. Currently, prospective pilot cohort studies are being conducted (ClinicalTrials.gov ID NCT06603506 and NCT07258186) to evaluate the feasibility and translatability of PDOs into routine clinical practice as a precision medicine tool. In one of these, Gall et al. aim to assess the efficacy of Poly(ADP-ribose) polymerase (PARP) inhibitors and validate the usefulness of PDOs in predicting clinical responses to treatment. Collectively, PDOs have shown substantial potential as a clinically relevant in vitro model for predicting sensitivity to some drugs as well as the clinical prognosis of patients. Ultimately, this will create avenues for the development of targeted fertility-sparing treatments using PDOs that will enable suitable subsets of women diagnosed with endometrial carcinoma to conceive and carry a successful pregnancy.

## 6. Modelling the Placenta

**Figure 3:**
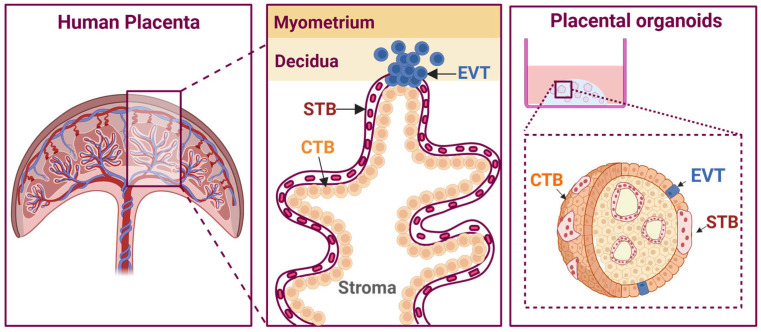
Schematic comparison of the human placenta and placental organoid models. The left panel illustrates the anatomical organisation of the human placenta, highlighting branching villous structures that extend into the intervillous space. The central panel depicts a magnified view of a placental villus, showing cytotrophoblasts (CTBs) underlying the multinucleated syncytiotrophoblast (STB) and the emergence of extravillous trophoblasts (EVTs) that invade through the decidua toward the maternal myometrium in order to remodel maternal spiral arteries and facilitate enhanced blood supply. The right panel demonstrates how placental organoids recapitulate key trophoblast lineages in vitro, forming spherical structures containing CTB progenitors, STB-like syncytial regions, and EVT-like cells. Together, these schematics illustrate how organoid systems model fundamental features of human placental architecture and trophoblast differentiation.

The placenta (Figure 3) is a critical organ required of healthy fetal development. Proper placental development requires a complex dialogue between fetal trophoblast cells, maternal endometrium, and the decidua. This culminates in a transient organ that is vital for nutrient, waste, and oxygen exchange between mother and fetus. Placental dysfunction contributes to pregnancy complications such as pre-eclampsia, fetal growth restriction, and pre-term labour, among others [125,126].

At 7 days post conception, the human blastocyst begins attachment to the endometrium via adhesion of the trophectoderm layer to a suitably receptive LE [126,127]. Following implantation, the outer trophectoderm layer begins to proliferate and invade into the maternal decidua. High surface area is essential for adequate gas exchange, nutrient transport, and waste disposal. This is achieved by the development of tree-like villi that occupy the intervillous space [128]. To anchor the placenta, villi invade into the decidua, whilst floating villi occupy the intervillous space surrounded by maternal blood [127,129]. Villi comprise different trophoblast subsets with discrete functions, including cytotrophoblasts (CTBs), syncytiotrophoblasts (STBs), and extravillous trophoblasts (EVTs). CTBs comprise the largest cell population and give rise to both STBs and EVTs (Figure 3) [130]. STBs have dual functions: (1) acting as the main site of exchange in the placenta and (2) secreting essential pregnancy hormones like human chorionic gonadotropins α and β (hCGα/β [130]. STBs are formed via the fusion of multiple CTBs into a single, multi-nucleated layer. EVTs are also derived from CTBs and invade into the decidua and remodel maternal spiral arteries to ensure there is adequate blood exchange. As each trophoblast cell type contributes in a distinct way to placental development and function, understanding their patterns of growth and the mechanisms that lead to their dysregulation provides important insight into both normal and abnormal placental biology.

### 6.1. Placental Cell Lines

Human choriocarcinoma cell lines such as BeWo, JEG-3, JAR, and HTR-8/SVneo are available to model trophoblast functions; however, these cells have low fusion rates and vary in function compared to primary trophoblast cells [131,132]. Typically, BeWo cells are used for syncytialisation studies, whereas others are used for invasive EVT studies [132]. Current in vitro models using primary CTBs are limited by their ethical and practical accessibility. Additionally, CTBs are difficult to maintain when cultured [132]. While animal models have provided insight into many pregnancy establishment processes, there are distinct differences in trophoblast function between rodents and humans. Two-dimensional trophoblast models have been reviewed elsewhere [126]. Therefore, improved models are required for significant advances in placental and reproductive research.

### 6.2. Trophoblast Organoids

Trophoblast organoids (TBOs) can be derived through three principal approaches: (1) culturing primary trophoblast cells isolated from first-trimester placental tissue (pTBOs); (2) culturing isolated trophoblast stem cells (tsc-TBOs); or (3) through isolation of term placental villi (Figure 3) [126]. Each method offers distinct advantages and limitations. Primary TBOs provide a valuable window into early placental development up to approximately 12 weeks of gestation. They exhibit robust expression of the proliferation marker Ki67 and can be maintained for up to 18 passages [133,134]. An additional strength of pTBOs is their preservation of patient-specific genetic backgrounds, enabling the modelling of particular genetic disorders [135]. tsc-TBOs are comparatively more accessible and amenable to transfection techniques, which are often challenging in primary cultures. Organoids derived from term placental tissue represent the most readily obtainable source and may also mitigate certain ethical considerations. Collectively, these derivation strategies offer researchers flexibility in selecting the model system most appropriate for addressing their specific scientific questions.

In 2018, Turco et al. (2018) and Haider et al. (2018) reported the generation of TBOs from first-trimester placental tissue [136,137]. These TBOs were derived from trophoblast cells isolated from 6–8-week placental tissue in a defined trophoblast organoid media (TOM) adapted from a previously defined media known to promote stemness and formation of organoids [138]. Additional factors included A83-01 to inhibit TGFβ signalling, Noggin to inhibit BMP signalling, epidermal growth factor (EGF), R-spondin to activate Wnt signalling, CHIR99021 as a GSK3α/β inhibitor, and prostaglandin E2. Within 2 weeks, 3D structures formed in Matrigel that were capable of being passaged and cryopreserved.

In both studies, trophoblast cells spontaneously formed villus-like structures in Matrigel and secreted key placental peptides, including human chorionic gonadotropin (hCG), growth differentiation factor 15 (GDF15), and pregnancy-specific glycoprotein [136,137]. Trophoblast identity was confirmed via expression of canonical markers GATA3, KRT7, EGFR, TFAP2A, and TFAP2C alongside the absence of HLA class I molecules. Villous cytotrophoblast identity was confirmed by apical expression of KRT7, EPCAM, and CDH1. Within the TBO, CTB cells readily fused to form multinucleated STB clusters, capable of secreting hCGβ [136]. Application of EVT medium promoted the migration of HLA-G+ cells out of the TBOs and Matrigel and adherence to the plastic beneath [136]. Trophoblasts were also capable of differentiation into HLA G-positive extravillous trophoblasts with demonstrable invasive capacity [136]. SCB identity was confirmed by expression of hCGβ, CD46, and CD71 [136]. Additionally, scanning electron microscopy revealed abundant secretory organelles and microvilli that were comparable to those seen in vivo [136]. Notably, the proliferative capacity of these organoids declined progressively with passaging and was reduced by passage 13, indicating that improved culture conditions may be required to sustain long term self-renewal. Nevertheless, molecular analyses identified a persistent CTB precursor population, suggesting that extended proliferative potential may be preserved under optimised conditions. Taken together, both Turco et al. and Haider et al. describe the generation of self-renewing 3D TBOs with the capacity to differentiate into mature trophoblastic lineages. These TBOs are comparable to trophoblast cells in vivo and provide a useful model in studying placental development and disease. Since then, Haider et al. has published an in-depth protocol, detailing how to enrich certain trophoblast subpopulations such as EVTs [139].

In the same year, Okae et al. (2018) established trophoblast stem cells (TSCs) from first-trimester placental tissue [140]. Trophoblast identity was confirmed via the human trophoblast identity criteria as proposed by Lee et al. (2016) [141], including expression of GATA3 and KRT7, among others. These TSCs could be maintained and expanded over numerous passages and demonstrated the capacity to differentiate into key trophoblast lineages, including STBs and EVTs.

Comparative analysis of p-TBOs and tsc-TBOs provides insights into conditions that influence model suitability. Both organoid systems satisfy key trophoblast identity criteria, including ELF5 promoter hypomethylation and expression of chromosome 19 microRNA cluster members [141]. A notable distinction, however, is the detection of HLA A/B expression in tsc-TBOs, which contrasts with the absence of these molecules in trophoblasts in vivo. Structural differences are also evident: tsc-TBOs contain fewer fused STBs and more closely resemble CTB cell columns at the tips of anchoring villi, whereas primary ones (p-TBOs) more faithfully recapitulate the villous architecture. Despite these differences, tsc-TBOs remain highly valuable for placental research, particularly because they are more amenable to genetic manipulation, including gene knockouts and plasmid transfections [126].

Karvas et al. reported the derivation of TBOs from naïve pluripotent human stem cells (hPSCs) [142]. These hPSC-derived TBOs closely resembled p-TBOs in tissue architecture, hormone secretion, and long-term self-renewal. Consistent with trophoblast identity, they expressed key markers such as GATA3, ELF5, and TFAP2C alongside downregulation of naïve and primed pluripotency markers, confirming successful lineage specification. Single-cell transcriptomic analysis further demonstrated that these organoids generated a spectrum of progenitor and differentiated trophoblast populations that parallel those observed in vivo. Moreover, similar to the findings of Turco et al. (2018), the hPSC-derived organoids could be directed to form EVT organoids when cultured in EVT-inducing medium originally defined by Turco and colleagues [136].

Unfortunately, term-placenta-derived TBOs have not been fully characterised. However, they do express appropriate trophoblast cell type markers, are capable of being modelled as apical-out, and are highly proliferative [133,143]. However, further studies are required regarding their suitability as a model for placental research.

Interestingly, TBOs show inverse polarity compared to in vivo placental structure with E-cadherin STBs in the centre of the TBOs. The E-cadherin-STB layer forms inside with the CTB layer on the apical surface, although the EVT population begins to migrate out of the organoid once differentiated [136,137]. Thus, these models are better suited to CTVB and EVT studies. Recently, Yang et al. generated ‘apical-out’ TBOs via suspended culture [144]. Epithelial organoid polarity has been reversed in other model systems [145,146,147]; thus, Yang et al. utilised these methods. TBOs were isolated from term placenta tissue and grown in Matrigel domes as described by Turco et al. in 2018 [136] and Haider et al. (2018) [137]. TBOs were ‘released’ from the Matrigel and cultured in low-attachment plates in an incubator with gentle rotation. Microscopy revealed hCGβ-positive staining on the outer layer with ITGA6-positive CTBs in the centre [144]. Luminex assays revealed that apical-out TBOs produced more hCGβ than their inverted counterparts, potentially attributed to an increased number of STBs or their secretion into the media being more accessible. Interestingly, unlike other defined TBO systems, apical-out TBOs showed reduced differentiation capacity into HLA-G+ EVTs. Unfortunately, once transferred to suspension, TBOs cannot be passaged further, impeding their ability to be cultured long-term [126]. Thus, apical-out TBOs provide a better-suited model for STB-specific studies, for example, infection and immunity.

Overall, while there have been advances in the field, a key limitation of currently available models remains a lack of cellular diversity. While trophoblasts dominate the first-trimester placenta, stromal, endothelial, and fetal immune cells also significantly contribute to placental development. A recent study by Huang et al. 2023 established placental villi organoids from patient tissue [148]. These organoids retained villi-like markers and were able to be differentiated into other placental cell types. Additionally, they retained endogenous immune cells, important as the placenta is an immune-privileged organ [149]. Establishing co-culture organoid systems that incorporate these additional cell types and their interactions with trophoblasts could enhance the physiological relevance of new models and reveal new insights into longstanding questions, such as how fetal-derived paracrine and endocrine signals regulate placental growth.

## 7. Models of Implantation

Modelling implantation presents substantial ethical and practical challenges. In 2023, Rawlings and colleagues generated an endometrial ‘assembloid’ to model embryo implantation in vitro [47,150]. Briefly, primary epithelial and stromal cells were expanded using established protocols and subsequently combined in a collagen hydrogel droplet and overlayed with standard EEO media supplemented with estradiol [31]. This coculture model is highly adaptable, as media composition can be altered to suit specific experimental aims. For example, to induce stromal decidualisation, cAMP and MPA can be added to the media. To model implantation, day-5 human blastocysts were placed onto pre-decidualised assembloids. Blastocysts readily expanded, and time-lapse microscopy revealed intimate communication between the trophectoderm, migratory decidual cells, and EEOs [47,150]. The authors claim the blastocysts were able to definitively attach to the EEOs inside the droplet; however, only one assembloid was assessed for this and could be an artefact of fixation. Additionally, trophectoderm attachment to the basal surface of EEOs is not expected given the reversed polarity of these structures in vivo. Similarly, confirmation that EEOs contain both luminal and glandular epithelium would create a model that mimics the in vivo environment more closely. Blastocyst viability was supported, as evidenced by hCG secretion 72 h after co-culture. However, senescent decidual cells contributed to matrix degradation, which could be mitigated by treatment with dasatinib, a potent inducer of decidualisation [150,151]. Overall, while promising, this assembloid model requires further optimisation to recapitulate the in vivo implantation environment more faithfully.

Kagawa et al. (2022) developed a human blastoid model capable of adhering to endometrial epithelial cells [66]. Blastoids were generated from naïve human pluripotent stem cells using previously defined protocols, yielding structures with a trophectoderm layer that expressed canonical markers such as GATA2, GATA3, and CDX2, demonstrated high proliferative capacity, and exhibited appropriate apical–basal polarity. Following confirmation of trophectoderm identity, blastoids were transferred onto a two-dimensional ‘open-faced endometrial layer’ produced by culturing established endometrial epithelial organoids (EEOs) in monolayer. Endometrial cells were hormonally primed with estradiol, progesterone, cAMP, and the Wnt inhibitor XAV939 to simulate the window of receptivity. Blastoids failed to attach to unprimed endometrial cells but adhered robustly to primed monolayers and induced local epithelial cell displacement, mirroring early events observed in utero. Within two days of co-culture, blastoids formed KRT7+ trophoblast cells that secreted hCGβ detectable by ELISA and commercial pregnancy tests [66]. A subset of cells further differentiated into STBs and EVTs confirmed via hCGβ and HLA-G expression, respectively. Some blastoids initiated formation of primitive pro-amniotic cavities and amnion-like structures, highlighting their potential for use in studying these tissue types. The blastoids remained stable and highly proliferative up until day 13, in which the experiment must be terminated ethically (14-day rule [152,153]). As with the model described by Rawlings et al., these blastoids were cultured on glandular rather than luminal epithelium, limiting their capacity to fully recapitulate the specific contributions of the luminal epithelium to embryo attachment and early placentation.

In 2024, Shibata et al. (2024) demonstrated the potential for a model that integrates the epithelial, stromal, embryonic, and vascular components of embryo implantation [154]. The researchers generated apical-out EEOs, which better recapitulate native tissue. The model included stromal cells and a self-formed endothelial network. Immunofluorescence staining showed a high similarity in the tissue architecture of this model when compared to in vivo tissue. Sn-RNA-seq. analysis revealed close similarity between them too. Both eSC-derived blastoids and 5–6 d.p.f. demonstrated the ability to adhere, although the latter was less successful, and implant into the EEO portions of the model. This included differentiation into trophoblast cell types by confirmation of SDC1 expression, a syncytiotrophoblast marker. Despite this, this model did not differentiate into other trophoblast lineages like EVTs. Additionally, hallmarks of embryonic development were not seen, like the appearance of the primitive streak or extraembryonic mesoderm. Further validation into the vascular phenotype was warranted as this was largely missing from this study.

Recently, Li et al. (2026), Molè et al. (2026), and Song et al. (2026) created 3D models of implantation containing the endometrial and embryo portion [155,156,157]. All three models were successful in modelling both the proliferative, secretive, and window of implantation of the cycle. Additionally, all the models demonstrated the ability of the embryo to differentiate into differentiated placental cell types. The Li et al. (2026) model utilises patient-derived endometrial tissue containing luminal and glandular epithelial and stroma [155]. A stromal cell core composed of stromal cells, Matrigel, and MMP-degradable hydrogel was placed followed by a layer of fragmented EEOs in a 50% Matrigel. These chips exhibited morphology that closely mimics in vivo tissue structure. On the other hand, the Molè et al. (2026) model, while also utilising patient-derived tissue, created a cell-engineered receptive endometrial scaffold technology, which they named CREST [156]. This model is derived from healthy patient tissue and contains luminal, epithelial, and stromal cell types. The stromal compartment was a hydrogel comprising dextran polymers, cyclodextrin crosslinker, and ECM materials found in the endometrium. EEOs were placed on top. In the Song et al. (2026) model, EEO Matrigel droplets were lightly punctured to create a small pocket where an embryo was transferred to [157]. In all models, EEOs underwent morphological change that mimicked the secretory-phase-like increased expression of cellular FOXA2 expression, PAEP secretion, pinopode bleb-like structures, and a tubular layout. Additionally, their models could support early implantation and development of human embryos up until 14 d.p.f. This was apparent in the development and differentiation of trophoblast cell types like CTBs, STBs, and EVTs that readily expressed lineage markers like CK7, CK8, hCG, SDC1, and HLA-G. Li et al. (2026) demonstrated the clinical impact of their model by deriving the same from patients with recurrent implantation failure [155]. Transcriptomic analysis revealed many DEGs that mimic those seen in vivo. Additionally, the authors demonstrated a marked decrease in the success of blastoids to adhere, implant, and invade the endometroid model from RIF models versus controls. A cocktail of FDA-approved compounds were applied to endometroids derived from RIF patients and demonstrated successful blastocyst development, including differentiation of trophoblastic cell types. This demonstrates clinical impact where this model could be used as a personalised medical approach for patients that suffer from RIF.

Because placentation is regulated by maternal immune populations, the incorporation of immune cells would enhance the physiological relevance of current in vitro implantation models. Immune cells not only promote fetal tolerance and protect against infection but also secrete key pregnancy-related factors that modulate trophoblast differentiation and invasion (reviewed elsewhere [158,159,160]). Taken together, these studies demonstrate that TBOs and TBO co-culture systems can successfully recapitulate several early events of human implantation, including blastocyst expansion, epithelial remodelling, and initial trophoblast differentiation. However, models remain limited by the absence of additional cellular contributors, most notably immune and endothelial cells, which play indispensable roles in vivo. Future iterations of these systems should therefore aim to integrate these components to more fully capture the cellular complexity and regulatory networks that govern early implantation and placental development.

## 8. Future Applications

The development of EEOs enables a wide range of future applications. EEOs provide a platform for drug screening studies aimed at treating endometrial disorders, while also allowing for assessment of potential off-target effects on endometrial tissue. Given that exogenous insults can impair fertility, suggested via direct disruption of endometrial function [161,162,163,164], such models offer a valuable tool for mechanistic investigation. Patient-derived EEOs are increasingly employed in oncology to evaluate the responsiveness of specific tumour types to chemotherapeutic agents [165], illustrating their potential to guide individualised therapeutic strategies.

Surprisingly since their inception in 2018, there are few published studies utilising TBOs for disease research. Karvas et al. (2022) describe deriving TBOs from naïve pluripotent stem cells (previously mentioned) and utilised these to investigate the viral infectivity of trophoblast cell types to SARS-CoV-2 [142]. Increased pregnancy complications have been reported in women who contract COVID-19 during pregnancy [166,167]. Interestingly, a subset of STB-like cells express ACE2 and TMPRSS2 (the two receptors used by SARS-CoV-2 [168]). However, infection with a SARS-CoV-2 isolate did not result in elevation of the associated spike protein, suggesting that the virus is unable to replicate efficiently inside this subset of cells. This provides an example of clinical application that could be applied to other diseases like syphilis, rubella, herpes, and others that cross the placenta in the first trimester [169].

The development of these methods opens the door to additional uses of tissue collected via chorionic villus samples (CVS). CVS procedures are used to obtain placental biopsies of ongoing pregnancy to screen for genetic disorders (such as Down syndrome and cystic fibrosis) with low rates of risk to the mother and fetus [170]. Considering these contain placental tissue, they could be utilised to derive patient-specific organoids. Similarly, this model can be used to compare differences between normal and disordered placentas. This would help investigate the role these cell types play in placenta diseases like preeclampsia, placenta accreta, choriocarcinomas, and even miscarriage. Additionally, specific genetic backgrounds can be used to derive TBOs, as Horii et al. demonstrated in the case of trisomy 21 [171]. Exogenous insults like pollutants, drugs, and alcohol can disrupt normal placental development and function [172,173,174]. Thus, generating TBOs from exposed placentae will provide insight into the mechanism of these substances. Likewise, exposing TBOs to these substances will help elucidate their mechanism of damage in a controlled environment.

To conclude, trophoblast organoids provide a rare opportunity to explore early placental development and could eventually recapitulate all stages of pregnancy in vitro. Ongoing research into first-trimester cellular behaviour has identified previously unrecognised cell populations that are now possible to model in culture. Although TSC-derived organoids have improved accessibility for researchers, primary trophoblast organoids still serve as the most faithful representation of the in vivo placenta.

Despite their growing value in research, these models still exhibit important limitations that must be considered. Comprising a mixed-cell population (glandular and luminal epithelia in EEOs and CTBs, STBs, and EVTs in PBOs) makes them inappropriate to model differences in these respective cell types. Additionally, EEOs between patients exhibit natural biological variation. However, this is beneficial when using these models in a personalised medicine approach, as they will be tailored to each patient. This has been demonstrated in endometroid models derived from RIF patients, mentioned previously.

## 9. Conclusions

Advances in three-dimensional organoid and co-culture technologies have fundamentally improved the capacity to model human endometrial biology, implantation, and placental development in vitro. Endometrial epithelial organoids (EEOs), particularly when combined with stromal, vascular, and microfluidic systems, overcome key limitations of animal models and two-dimensional cultures by recapitulating human-specific features such as cyclic hormone responsiveness, decidualisation, and epithelial–stromal crosstalk. The development of chemically defined and semi-synthetic matrices has further enabled long-term culture, reproducibility, and mechanistic interrogation of endometrial function in both health and disease.

Despite this progress, major challenges remain, including incomplete modelling of the luminal epithelium, unresolved issues of epithelial polarity, and limited incorporation of additional cellular compartments such as immune and endothelial cells. Addressing these gaps is critical for accurately defining the mechanisms underlying endometrial receptivity and implantation failure. In parallel, trophoblast organoids have provided unprecedented access to early placental development, faithfully modelling key trophoblast lineages and functions, although their physiological relevance will be further enhanced by incorporating supporting maternal and fetal cell types.

Importantly, patient-derived organoid systems have proven highly valuable for modelling gynaecological diseases associated with infertility, capturing disease heterogeneity, altered hormone signalling, and dysregulated cell–cell communication. Together, these evolving platforms offer powerful opportunities for mechanistic discovery like embryo attachment, glandular development, and early- and late-stage placentation. Personalised medicine and therapeutic screening will provide insights into the aetiology and interventions into gynaecological diseases like endometriosis, preeclampsia, and infertility, that can be tailored to each patient.

## Figures and Tables

**Table 1:** Components of human EEO culture medium.

Expansion Medium Components ^a^	Author								
	Boretto et al. (2017) [32]	Turco et al. (2017) [31]	Boretto et al. (2019) [34]		Haider et al. (2019) [35]	Fitzgerald et al. (2019) [36]	Luddi et al. (2020) [37]	Fitzgerald et al. (2023) [38]	Jiang et al. (2024) [39]
			EM-O EUT-O ECT-O HYP-O	EC-O					
WNT3A ^b^	+	+	-	-	-	-	-	-	-
EGF	+	+	+	+	+	+	+	+	+
HGF	-	+	-	+	-	-	+	+	+
NICO	+	+	+	+	-	+	+	+	+
FGF-10	+	+	+	-	-	+	+	+	+
ITS	+	-	+	-	-	+	+	+	+
NOG	+	+	+	+	+	+	+	+	+
RSPO	+	+	+	+	+	+	+	+	+
A83-01	+	+	+	+	+	+	+	+	+
N2	+	+	+	+	+	+	+	+	+
B27	+	+	+	+	+	+	+	+	+
NAC	+	+	+	+	-	+	+	+	+
Y-27632 ^c^	+	+	+	+	+	+	-	+	-
Others	E2 ^d^	SB202190	E2 ^d^, bFGF, SB202190	E2 ^d^, IL-6 ^e^, IGF-1, Lipid, SB202190	CHIR99021, PGE2	-	-	-	-
Hormones ^f^	E2, P4	E2, P4, cAMP, PRL, hCG, hPL	-	-	E2	E2, MPA, cAMP	E2, P4, cAMP	E2, MPA, PGE2 ^g^	E2, P4, cAMP

EM-O: healthy endometrial organoid, EUT-O: endometriosis eutopic endometrial organoid, ECT-O: endometriosis ectopic/lesion organoid, HYP-O: endometrial hyperplasia organoid, EC-O: endometrial cancer organoid, EGF: epidermal growth factor, HGF: hepatocyte growth factor, FGF-10: fibroblast growth factor 10, ITS: insulin-transferrin-selenium, NOG: Noggin-conditioned media, RSPO: human R spondin-1conditioned media, A83-01: ALK-4, -5, -7 inhibitor (TGFβ receptor inhibitor), NAC: N-acetyl L-cysteine, Y-27632: ROCK signalling inhibitor, E2: 17β-estradiol, SB202190: p38 MAPK inhibitor, bFGF: basic fibroblast growth factor, IL-6: interleukin 6, IGF-1: insulin-like growth factor 1, Lipid: chemically defined lipid concentrate, CHIR99021: glycogen synthase kinase 3β inhibitor, PGE2: prostaglandin E2, P4: progesterone, cAMP: 8-bromoadenosine 3′5′-cyclic monophosphate, PRL: prolactin, hCG: human chorionic gonadotropin, hPL: human placental lactogen, MPA: medroxyprogesterone acetate (synthetic, non-metabolisable form of P4). ^a^ Basal medium components also include DMEM/F12, glutamine and antibiotics. ^b^ Initially used for mouse EEO media, not needed for optimised human EEO media. ^c^ Only for organoid formation or after dissociation for passaging. ^d^ Only for organoid expansion. ^e^ Not essential. ^f^ Used for EEO differentiation and assessing steroid hormone responsiveness. ^g^ Growth factors removed from medium for hormone treating.

**Table 2:** Comparison of 3D matrices for human EEO culture.

Matrix	Key Features	Advantages	Limitations	References
Matrigel	ECM hydrogel extracted from Engelbreth–Holm–Swarm (EHS) mouse sarcoma rich in laminin, collagen IV, proteoglycans and growth factors; thermally gelates at ~37 °C.	Supports efficient formation and long-term growth of EEOs, replicating gland-like structure. Provides biochemical cues and structural support that mimic native basement membrane. Widely adopted “gold-standard” scaffold for 3D organoid culture across tissues.	Poorly defined and variable composition; batch-to-batch differences impact reproducibility. Murine origin raises xenogeneic concerns and limits clinical translation. Contains endogenous growth factors that may confound mechanistic studies; requires growth-factor-reduced formulation. Unsuitable for stable epithelial–stromal co-culturing of endometrial epithelial and stromal cells.	[31,32,34,35,36,37,38,51]
Basement membrane extract (BME), e.g., Cultrex, Geltrex	EHS mouse-sarcoma-derived basement membrane extract similar to Matrigel.	Similar biological composition to Matrigel. Supports organoid growth across epithelial tissues. Can be used interchangeably with Matrigel.	Same origin and undefined composition issues as Matrigel. Less widely used in standard EEO culture (most studies use Matrigel).	[39,52]
Collagen I (natural hydrogel)	Fibrillar collagen hydrogel reflecting predominant ECM protein.	More defined than complex BMEs. Mechanical properties can be tuned. Supports endometrial epithelial and stromal co-cultures and assembloids.	Susceptible to cell-mediated contraction over long-term culture. Lacks native basement membrane signals (laminin, etc.) unless supplemented.	[43,47,51,53]
Decellularised tissue ECM hydrogels	Hydrogel derived from decellularised endometrial tissue ECM (tissue-specific ECM).	Tissue-mimetic biochemical composition providing native cues. May better recapitulate in vivo microenvironments than other ECMs.	Complex and variable composition; decellularisation efficiency affects reproducibility. Less widely standardised in endometrial models currently.	[49]
Synthetic ECM (e.g., PEG-based with adhesion peptides)	Fully defined synthetic hydrogel functionalised with cell-adhesion motifs (e.g., GFOGER, RGD) and tuned mechanics.	Chemically defined, reproducible, and xeno-free. Supports co-culture of endometrial epithelial and stromal cells in organoid/assembloid models. Permits tuning of stiffness and ligand presentation to mimic native endometrial biomechanical environment.	Development can be complex; requires careful optimisation of biochemical cues for cell attachment. Some synthetic matrices may lack full spectrum of native ECM signals unless engineered in.	[46]
Hybrid/semi-synthetic hydrogels	Hybrids combining natural and synthetic components (e.g., GelMA + ECM proteins).	Aims to integrate biological cues of natural ECM with tunability of synthetic materials. Supports stable endometrial epithelial–stromal co-culture.	Complexity in formulation and reproducibility. Performance in endometrial-specific contexts still emerging.	[50]
Scaffold-free or agarose/micromold approaches	Non-ECM physical scaffolds (e.g., micromolded agarose) to spatially organise organoids or assembloids.	Useful for microarchitectural control, spheroid agglomeration, or support when matrix cues are provided by cells.	Does not provide biochemical ECM cues on its own; often combined with other matrices.	[54]

BME: basement membrane extract, ECM: extracellular matrix, PEG: polyethylene glycol, GelMA: gelatin methacryloyl.

## Data Availability

No new data were created or analysed in this study.
